# Oral Potassium Malabsorption Following Bariatric Surgery

**DOI:** 10.7759/cureus.28607

**Published:** 2022-08-30

**Authors:** Neslida Kodra, Raphael Khella, Brian G Nudelman, Bryan Dawkins

**Affiliations:** 1 Medicine, Dr. Kiran C. Patel College of Osteopathic Medicine, Fort Lauderdale, USA; 2 Family Medicine, Lakeside Medical Center, Belle Glade, USA

**Keywords:** roux-en-y, potassium replenishment, gastric bypass complications, potassium malabsorption, gastric bypass surgery

## Abstract

Bariatric surgery is one of the most effective long-term solutions for treating obesity due to its sustained weight loss and reduction of obesity-related comorbidities. However, nutritional deficiencies are common due to the alteration of the anatomy and physiology of the gastrointestinal tract. These include the malabsorption of macronutrients, vitamins, minerals, trace elements, and drugs. In this report, we present the case of a female patient who underwent Roux-en-Y gastric bypass surgery and subsequently developed exclusive potassium malabsorption refractory to oral replenishment.

## Introduction

Bariatric surgery has been a popular and widely studied method of weight loss within the last three decades, with approximately two million patients undergoing surgery between 1993 and 2016 [1]. It improves the long-term quality of life in obese patients through sustained weight loss, reduction of obesity-associated cardiovascular risk by 42%, and improving glycemic control [1, 2]. Therefore, it is currently the most effective therapy available for patients with a body mass index of ≥ 40 or ≥ 35 kg/m2 with co-morbidities [2]. Bariatric surgery influences weight loss through three key mechanisms including caloric restriction, nutrient malabsorption, and/or a combination of the former two methods [2]. In recent years, the two most common procedures are the Roux-en-Y gastric bypass and the sleeve gastrectomy [3]. The surgery consists of the creation of a small proximal gastric pouch that is separated from the distal stomach and anastomosed to a limb of the small bowel. Gastric bypass primarily influences weight loss via caloric restriction due to the newly formed gastric pouch; additionally, weight loss can be attributed to hormone inhibition when the foregut is bypassed. A major factor that can contribute to the success of the surgery, as well as create various complications, is the length of the Roux (small intestine) limb, with approximately 150 cm being the maximum length that most surgeons will aim for [4]. The longer the Roux limb, the shorter the common limb, which is where digestion and absorption of important nutrients occur. Malabsorption and maldigestion are important contributors to nutritional deficiencies that present post-bariatric surgery. While reported incidences vary widely, the most common nutritional issues following bariatric surgery are iron, vitamin B12, or folate- deficiency anemia (33% to 49% incidence), calcium deficiency (10% incidence), and vitamin D deficiency (25% to 73% incidence) [2]. Other common deficiencies include fat-soluble vitamins (A, E, and K), thiamine (B1), and minerals (magnesium, zinc, copper, and selenium) [2]. Potassium deficiency, on the other hand, is a less reported and not as widely studied complication of gastric bypass surgery. 

## Case presentation

Our patient is a 42-year-old female who was evaluated in the inpatient setting four weeks post-Roux-en-Y gastric bypass surgery. Her medical history is significant for diabetes mellitus type 2, hypertension, endometrial hyperplasia, and thyroidectomy secondary to malignancy. The patient required bariatric surgery because she was too obese to undergo a hysterectomy. Just before surgery, she weighed 143 kgs with a body mass index (BMI) of 57.7. Post-surgical weight loss was 30 kgs in four weeks. Following bariatric surgery, she remained on a full liquid diet at home and supplemented with daily multivitamins. 

Four weeks post-surgery, however, she developed abdominal pain, vomiting, and weakness with the inability to ambulate. She was admitted to our intensive care unit for diabetic ketoacidosis and severe hypokalemia of 2.2 mmol/L. She also had concurrent hypophosphatemia at 1.8 mmol/L. She was started on basal and bolus insulin and intravenous (IV) potassium. An insulin drip was not administered due to hypokalemia. The patient was unable to tolerate the burning sensation from the IV potassium, as well as the frequent loose stools that began within 30 to 60 minutes of administration, and was subsequently switched to oral potassium. Over the next four days of hospitalization, the patient’s glucose levels returned to baseline, however, she remained hypokalemic with serum potassium levels ranging from 2.6 to 3.0 mmol/L. Serum magnesium levels were within normal limits. The oral potassium was not absorbed and she was able to identify the intact potassium tablets in her stool. The patient revealed that she had previously experienced a similar episode of hypokalemia refractory to oral supplementation just two weeks post-bariatric surgery. She recalled passing intact potassium tablets in her stool during that episode as well. Due to concerns of malabsorption, the patient was subsequently switched to a potassium powder mixed with water, and despite experiencing loose stools, which were treated with loperamide, she was able to achieve an acceptable potassium level of 3.5 mmol/L. 

## Discussion

The main issue presented in this case was the malabsorption of oral potassium post-gastric bypass surgery. Due to the nature of this surgery, it is very common for patients to develop malabsorption and maldigestion. The restructuring of the gastrointestinal tract causes decreased mixing of enzymes secreted from the pancreas and gallbladder thus impairing breakdown [5]. While it is widely known that bariatric surgery can cause nutritional deficiencies, this patient’s presentation was unique in that she was exclusively deficient in potassium and her hypokalemia was refractory to oral supplementation specifically. There was a multitude of factors in this patient’s hospital course that may have contributed to her hypokalemia including vomiting, diarrhea, and diabetic ketoacidosis. Prior to admission, she may have also been hypokalemic due to dehydration, poor oral intake, and rapid weight loss. However, our inability to correct her hypokalemia with oral potassium supplementation and the passing of undigested pills were concerning for underlying malabsorption. Potassium is primarily absorbed in the duodenum and jejunum. Because Roux-en-Y surgery decreases the length of the common limb of the small bowel, there is effectively decreased surface area for absorption [4]. Additionally, we hypothesized that the potassium pill itself was not broken down due to her newly decreased concentration of digestive enzymes post-bypass. The powdered form was more effective because it is absorbed through the lining of the small intestine at a faster rate and bypasses the need for the breakdown of a pill or capsule. A 2021 cross-sectional study found that bariatric surgery reduces the time to maximal plasma concentration (Tmax) and the maximum plasma concentration (Cmax) of most drugs and vitamins [6]. Therefore, while the small intestine is able to absorb the drug more rapidly, it ultimately absorbs less of it, thus significantly decreasing serum drug concentration [6]. In our patient’s case, her Cmax for absorption of the oral potassium pill was likely similarly decreased. 

## Conclusions

This case outlines a presentation of oral potassium malabsorption in a patient with hypokalemia refractory to oral replenishment post bariatric surgery. Despite the many benefits of bariatric surgery, major complications related to malabsorption can occur and patients may present with subsequent nutritional deficiencies that can be refractory to traditional therapies. Importance should be placed on monitoring for deficiencies through standard blood work during scheduled post-operative visits. While routine blood work can identify asymptomatic hypokalemia, levels should always be assessed immediately after a patient develops any symptoms, which can range from mild fatigue and muscle cramps to severe heart palpitations and arrhythmias. Once identified, employing appropriate treatment via oral or parenteral therapy is crucial. Severe potassium deficiencies can be life-threatening and require hospitalization where levels are monitored throughout treatment to ensure proper absorption. Therefore, early detection and treatment of potassium deficiencies are essential in minimizing consequences to patients post-surgery. 
